# Cholecystectomy, Conversion and Complications

**DOI:** 10.1155/2000/56760

**Published:** 2000-08

**Authors:** M. H. Thompson, J. R. Benger

**Affiliations:** The Department of Surgery Southmead Hospital Bristol BSIO 5NB United Kingdom

## Abstract

Background Faced with a difficult laparoscopic cholecystectomy the surgeon may feel that conversion to open operation would risk greater complications because of the laparotomy. Information on the effect of conversion is lacking. The purpose of this study is to measure the complications of laparoscopic cholecystectomy and observe the effect of the conversion rate.

Methods A total of 957 patients were studied. There were three consecutive series of patients; the first undergoing open cholecystectomy (384 patients), the second laparoscopic cholecystectomy with a 5.8% conversion rate (412 patients) and the third laparoscopic cholecystectomy with a 1.3% conversion rate (161 patients). Data was collected prospectively using a continuous audit, and the complication rate compared on an intention to treat basis. In addition a panel of experienced surgeons was asked to score the complications depending on their severity and a composite complication score calculated. Comparison between the 3 groups was then undertaken. Results Open cholecystectomy produced a postoperative complication rate of 6%. Initially this appeared to fall to 3.1% with the introduction of laparoscopic cholecystectomy, but when the complications occurring in the converted patients were included (*i.e.,* on an intention to treat basis) the rate increased to 5.6% in the first group of laparoscopically- treated patients and 3.1% in the second. These differences were not statistically significant. A similar pattern emerged when scoring the severity of the complications as judged by the expert panel. The inclusion of intra-operative complications appears to remove any small advantage for laparoscopic cholecystectomy. The reduction in the conversion rate between the two laparoscopic groups from 5.8% to 1.2% was statistically significant.

Conclusion When considered on an intention to treat basis laparoscopic cholecystectomy offers no advantage over open operation in terms of the frequency or severity of complications. Reducing the frequency of conversion from a laparoscopic to an open procedure also has no significant effect on the complications encountered. We conclude, therefore, that the complication rate is independent of the conversion rate and that the surgeon, when faced with difficulty at laparoscopic cholecystectomy, should not be deterred from converting to open operation for fear of the post-operative consequences.


*HPB Surgery*, 2000, Vol. 11, pp. 373–378
Reprints available directly from the publisher
Photocopying permitted by license only

© 2000 OPA (Overseas Publishers Association) N.V.
Published by license under
the Harwood Academic Publishers imprint,
part of The Gordon and Breach Publishing Group.
Printed in Malaysia.

### Cholecystectomy, Conversion and Complications

M. H. THOMPSON* and J. R. BENGER

*The Department of Surgery, Southmead Hospital, Bristol BS10 5NB, United Kingdom*

*(Received 19 March 1999)*

**Background** Faced with a difficult laparoscopic cholecystectomy the surgeon may feel that conversion to open operation would risk greater complications because of the laparotomy. Information on the effect of conversion is lacking. The purpose of this study is to measure the complications of laparoscopic cholecystectomy and observe the effect of the conversion rate.

**Methods** A total of 957 patients were studied. There were three consecutive series of patients; the first undergoing open cholecystectomy (384 patients), the second laparoscopic cholecystectomy with a 5.8% conversion rate (412 patients) and the third laparoscopic cholecystectomy with a 1.3% conversion rate (161 patients). Data was collected prospectively using a continuous audit, and the complication rate compared on an intention to treat basis. In addition a panel of experienced surgeons was asked to score the complications depending on their severity and a composite complication score calculated. Comparison between the 3 groups was then undertaken.

**Results** Open cholecystectomy produced a post-operative complication rate of 6%. Initially this appeared to fall to 3.1% with the introduction of laparoscopic cholecystectomy, but when the complications occurring in the converted patients were included (*i.e.*, on an intention to treat basis) the rate increased to 5.6% in the first group of laparoscopically-treated patients and 3.1% in the second. These differences were not statistically significant. A similar pattern emerged when scoring the severity of the complications as judged by the expert panel. The inclusion of intra-operative complications appears to remove any small advantage for laparoscopic cholecystectomy. The reduction in the conversion rate between the two laparoscopic groups from 5.8% to 1.2% was statistically significant.

**Conclusion** When considered on an intention to treat basis laparoscopic cholecystectomy offers no advantage over open operation in terms of the frequency or severity of complications. Reducing the frequency of conversion from a laparoscopic to an open procedure also has no significant effect on the complications encountered. We conclude, therefore, that the complication rate is independent of the conversion rate and that the surgeon, when faced with difficulty at laparoscopic cholecystectomy, should not be deterred from converting to open operation for fear of the post-operative consequences.

*Keywords:* Cholecystectomy, laparoscopy, complications, conversion

### INTRODUCTION

The decision to convert from laparoscopic to open cholecystectomy may not be easy. The surgeon may feel that conversion may induce more complications and delay a judicious conversion. There is no information in the literature to help in this respect. We have, therefore, compared the results of three groups of patients undergoing cholecystectomy: one open operation

*Corresponding author. Tel.: 0117 959 6163, Fax: 0117 9595168.

to act as the previous standard and the other two laparoscopic cholecystectomy with two different rates of conversion. The comparison with the results of open cholecystectomy is necessary to establish the results on an intention to treat basis.

The results of treatment on an intention-to-treat basis has only been described in two of eleven previous series of laparoscopic cholecystectomy [1–11] but without comparison with open cholecystectomy. It is not given in four randomised trials [12–15], one meta-analysis [16], two large community studies [17], two reviews of complications [19, 20] and two single centre observational studies [19, 21].

There are difficulties measuring complications after laparoscopic cholecystectomy. They are usually given as a list and quoted as a percentage. Sometimes, they are classified as minor or major depending on whether the hospital stay was prolonged as a result. After laparoscopic surgery this may be invalid as a patient can be discharged only to return a few days later with the complication. These difficulties were embraced by Clavien [22] who proposed a four-grade system depending on the degree of threat to life, residual disability and threat to life. This system, however, is open to interpretation. We therefore devised a different system. A panel of experts was asked to score each of the complications so that numerical comparisons between the groups could be made. The concept involved is that the panellists would include all the variables when deriving their score.

### METHODS

Patients undergoing cholecystectomy under the care of one surgeon were divided into three groups:

*Group 1* 384 consecutive patients undergoing open cholecystectomy.

*Group 2* 412 consecutive patients undergoing cholecystectomy with the intention to treat laparoscopically, with 24 converted to open operation (5.8%). In this period 16 patients intentionally underwent an open cholecystectomy; 3 for empyema, 4 for cholangiography, 2 because of extensive previous surgery, 4 in conjunction with another operation, 1 because of pregnancy and 2 with peritonitis.

*Group 3* 161 consecutive patients, treated subsequently to those in Group 2, also undergoing cholecystectomy with an intention to treat laparoscopically. Two of these were converted (1.2%).

No patient in group three was intentionally treated using open cholecystectomy. Patients with bile duct stones were excluded from the study.

Open cholecystectomy was performed through a transverse upper abdominal incision confined, if possible, to the rectus sheath. Laparoscopic cholecystectomy was carried out using a four-port "American" technique. Single-dose antibiotic prophylaxis was used throughout. All patients wore compression stockings and those at high risk of venous thromboembolism were given subcutaneous heparin. Operations were performed by a consultant, experienced trainees with or without consultant supervision or junior trainees with consultant supervision.

Data about complications were collected in a prospective audit recorded during and immediately after the patient's hospital stay. Complications have been divided into peri- and post-operative groups.

In order to construct a complication "score" 11 experienced surgeons were presented with the list of complications that had occurred and were requested to give a score to each complication on a scale of 1–10; ten being the most severe. A mean score for each complication was calculated and from this a score per 100 cases derived so that the three groups of patients could be compared. The detail of the scoring

TABLE I The complications of cholecystectomy and the scores given to them by an expert panel of surgeons

| Panellist | 1 | 2 | 3 | 4 | 5 | 6 | 7 | 8 | 9 | 10 | 11 | Mean |
|---|---|---|---|---|---|---|---|---|---|---|---|---|
| Bile leak | 2 | 4 | 5 | 7 | 3 | 5 | 5 | 4 | 7 | 5 | 3 | 4.5 |
| Paralytic ileus | 6 | 4 | 5 | 3 | 2 | 6 | 5 | 3 | 2 | 2 | 3 | 3.7 |
| Abdominal pain | 1 | 3 | 4 | 7 | 1 | 6 | 4 | 4 | 2 | 2 | 3 | 3.4 |
| Wound infection | 2 | 2 | 3 | 2 | 2 | 1 | 5 | 5 | 3 | 3 | 2 | 2.7 |
| Wound dehiscence | 8 | 7 | 6 | 10 | 8 | 8 | 6 | 8 | 4 | 8 | 5 | 7.1 |
| Abdominal abscess | 8 | 6 | 8 | 8 | 4 | 9 | 10 | 9 | 10 | 8 | 5 | 7.7 |
| Urinary tract infection | 1 | 1 | 3 | 1 | 1 | 3 | 3 | 4 | 3 | 3 | 2 | 2.3 |
| Retention of urine | 2 | 1 | 4 | 1 | 1 | 2 | 3 | 1 | 3 | 2 | 1 | 1.9 |
| Haematemesis | 5 | 5 | 5 | 6 | 1 | 9 | 8 | 8 | 4 | 4 | 4 | 5.4 |
| Chest infection | 2 | 1 | 5 | 1 | 1 | 1 | 5 | 3 | 4 | 5 | 2 | 2.7 |
| Pleural effusion | 5 | 5 | 3 | 6 | 2 | 4 | 5 | 3 | 3 | 2 | 3 | 3.5 |
| Deep venous thrombosis | 3 | 5 | 5 | 7 | 2 | 5 | 7 | 4 | 5 | 3 | 4 | 4.5 |
| Pulmonary embolus | 9 | 7 | 10 | 10 | 3 | 8 | 10 | 6 | 10 | 8 | 5 | 7.8 |
| Stroke | 10 | 9 | 9 | 9 | 3 | 10 | 10 | 9 | 10 | 8 | 5 | 8.4 |
| Myocardial infarction | 9 | 9 | 9 | 8 | 3 | 10 | 10 | 9 | 10 | 8 | 6 | 8.3 |
| C. difficile infection | 8 | 6 | 3 | 6 | 3 | 6 | 4 | 6 | 4 | 4 | 4 | 4.9 |

system derived from the expert panel is given in Table I.

### RESULTS

Twenty-four of the 412 patients in Group 2 (5.8%) and 2 out of the 161 (1.8%) patients in Group 3 underwent conversion to open operation. This reduction in the conversion rate between Groups 2 and 3 was statistically significant (chi-squared, $p = 0.03$).

The post-operative complication rate for open cholecystectomy (Group 1) was 6%. In the first series of laparoscopic cholecystectomy (Group 2) it fell to 3.1% for those completed laparoscopically, but the patients converted to open operation suffered a complication rate of 33%. When combined the overall complication rate for group two on an intention-to-treat basis was 5.6%. The post-operative complication rate for patients in Group 3 (laparoscopic cholecystectomy with a lower conversion rate) was 3.1%; there were no complications in the two patients converted to open operation. There is no statistically significant difference between these figures for the three groups. Further details will be found in Table II.

The scores for severity of post-operative complications derived from the panel of experts, were 23, 20 and 16 for Groups 1, 2 and 3 respectively. These are the scores on an intention-to-treat basis (including the converted cases), and are given per 100 cases.

The complications that occurred are detailed in Table II. There was a marked change in the nature of the complications during the course of the study, with *Clostridium* difficile infection being the most common complication in the later cases treated laparoscopically. This occurred only in patients who had been admitted to hospital with an acute attack of cholecystitis treated with antibiotics just before surgery.

Three severe intra-operative complications occurred. The bile duct was transected at open cholecystectomy: it was recognised and repaired without subsequent morbidity. At laparoscopic cholecystectomy the small bowel was entered with a trocar in one case: it was recognised and repaired without morbidity. In another patient, profound bradycardia leading quickly to asystole occurred: this responded to pharmacological treatment although a short spell of cardiac massage was required: this operation was also completed uneventfully. The result of adding these intra-operative complications to the figures for post-operative complications outlined above is detailed in Table III. There is now no advantage for the laparoscopic approach: the overall complication

TABLE II The complications of cholecystectomy

| | Group 1 | Group 2 (Laparoscopic) | | Group 3 (Laparoscopic) | |
|---|---|---|---|---|---|
| Complication | Open | Complete | Converted | Complete | Converted |
| Bile leak | – | 4 | 2 | – | – |
| Paralytic ileus | – | 2 | – | – | – |
| Abdominal pain | – | – | – | 1 | – |
| Wound infection | 3 | – | – | – | – |
| Wound dehiscence | 1 | – | – | – | – |
| Abdominal abscess | 1 | 1 | – | – | – |
| Urinary tract infection | 1 | – | – | – | – |
| Retention of urine | 5 | 1 | 3 | – | – |
| Haematemesis | – | 1 | – | – | – |
| Chest infection | 6 | 1 | 1 | 1 | – |
| Pleural effusion | 1 | – | – | – | – |
| Deep venous thrombosis | 2 | 1 | 1 | – | – |
| Pulmonary embolus | 2 | – | – | – | – |
| Stroke | 1 | – | – | – | – |
| Myocardial infarction | – | 1 | – | 1 | – |
| C. difficile infection | – | – | 1 | 3 | – |
| Complication rate % | 6 | 3 | 33 | 3 | 0 |
| Score per 100 cases | 23 | 14 | 112 | 16 | 0 |

TABLE III Panel scores for intra- and post-operative complications combined

| Group | Complication score per 100 cases |
|---|---|
| Group 1 (Open) | 25 |
| Group 2 (Laparoscopic) | 20 |
| Group 3 (Laparoscopic) | 25 |

scores are significantly worse for the patients in Group 3, despite the lower conversion rate.

### DISCUSSION

The first finding in this study was the inability of the laparoscopic approach to reduce the complication rate of cholecystectomy. Although, at first sight, it appears that laparoscopic cholecystectomy halved the complication rate, inclusion of the converted cases restored the rate to that for the open operation at 6%.

Secondly, our results show that a statistically significant reduction in the conversion rate was not accompanied by an equivalent reduction in the complication rate for either of the two scoring systems used (Fig. 1). This is surprising as these are consecutive cases. The improvements in perioperative care with time would be expected to produce better results but this has not been observed. These findings are at variance with those of Jatzko *et al.* [21] and to a lesser extent with Williams *et al.* [22], although the latter study did not include patients with acute cholecystitis in the laparoscopic group.

The present study shows a striking change in the nature of the complications seen. Bile leaks of a minor nature, which spontaneously settled, occurred in the early part of the laparoscopic group, and we suspect that this was due to over zealous cleaning of the cystic duct since this is the only change in technique which might explain the results. It would seem reasonable to expect that, having seen this complication disappear, the complication rate and score would reduce but other complications have emerged to take its place. There were two clear perioperative laparoscopic complications: bowel injury and a profound bradycardia shortly after establishment of the pneumoperitoneum, which rapidly progressed to asystole. Both operations were completed uneventfully. When these cases are taken into consideration we were not able to demonstrate an overall reduction in the rate of complications of laparoscopic cholecystectomy on an intention to treat basis.

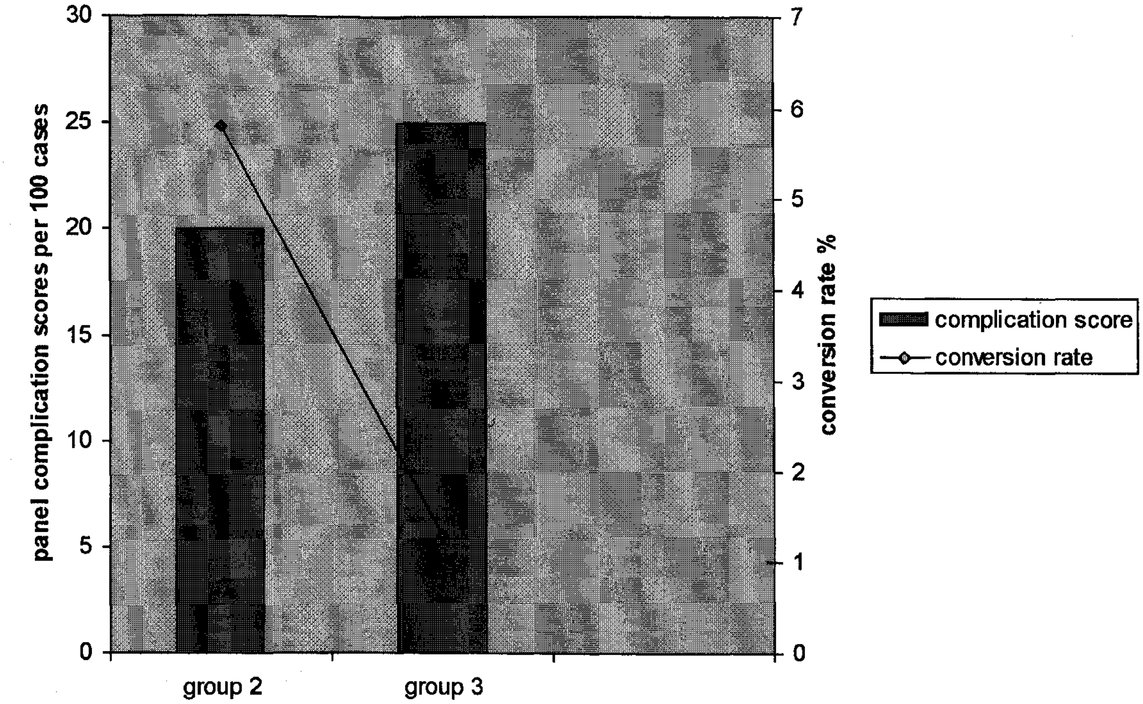

FIGURE 1 The relationship between conversion rate and complication scores.

The slight, and statistically insignificant, reduction in post operative complications associated with a lower conversion rate is not sufficient to prevent the surgeon from converting a laparoscopic to an open operation when faced with difficulty. It is justifiably better to convert to an open operation than to risk a bile duct injury.

### Composition of the Panel of Experienced Surgeons Contributing to the Scoring System for Complications

Professor D. Alderson, Bristol Royal Infirmary, Bristol: Mr. C. P. Armstrong, Frenchay Hospital, Bristol: Mr. D. C. Britton, Royal United Hospital, Bath: Mr. B. J. Britton, John Radcliffe Hospital Oxford: Mr. I. A. Eyre-Brook, Musgrove Park Hospital, Taunton: Mr. J. W. L. Fielding, Queen Elizabeth's Hospital, Birmingham: Mr. M. W. L. Gear, Gloucester Royal Hospital, Gloucester. Mr. R. H. Kennedy, Yeovil Hospital, Yeovil: Mr. M. P. McGinn, Southampton General Hospital, Southampton. Mr. B. Rees, University Hospital of Wales, Cardiff: Dr. N. J. Soper, Barnes Hospital, St. Louis, USA.

### *Acknowledgments*

The authors wish to extend their grateful thanks to the panel of surgeons.

### *References*

[1] Wherry, D. C., Rob, C. G., Marohn, M. R. and Rich, N. M. (1994). An external audit of laparoscopic cholecystectomy performed in medical treatment facilities of the Department of Defense. *Ann. Surg.*, **220**, 626–34.

[2] Cushieri, A., Dubois, F., Mouiel, J., Mouret, P., Becker, H., Buess, G., Trede, M. and Troidl, H. (1991). The European experience with laparoscopic cholecystectomy. *Am. J. Surg.*, **161**, 385–387.

[3] Bailey, R. W., Zucker, K. A., Flowers, J. L., Scovill, W. A., Graham, S. M. and Imbembo, A. L. (1991). Laparoscopic Cholecystectomy. Experience with 375 consecutive cases. *Ann. Surg.*, **214**, 531–41.

[4] Troidl, H., Spangenberger, W., Langen, R., Al-Jaziri, A., Eyspach, E., Neugebauer, E. and Dietrich, J., Laparoscopic Cholecystectomy: Technical performance, safety and patients benefit. *Endoscopy*, **24**, 252–26 1.

[5] Voyles, C. R., Petro, A. B., Meena, A. L., Haick, A. J. and Koury, A. M. (1991). A practical approach to laparoscopic cholecystectomy. *Am. J. Surg.*, **161**, 365–370.

[6] Berci, G. and Sackier, J. M. (1991). The Los Angeles experience with laparoscopic cholecystectomy. *Am. J. Surg.*, **161**, 382–384.

[7] Graves, H. A., Ballinger, J. F. and Anderson, W. J. (1991). Appraisal of laparoscopic cholecystectomy. *Ann. Surg.*, **213**, 655–664.

[8] The Southern Surgeons Club. A prospective analysis of 1518 Laparoscopic cholecystectomies. *New Eng. J. Med.* (1991), **324**, 1073–1078.

[9] Birdi, I., Armon, M., Hunt, T. M., Jervis, P., Veitch, P. S. and Barr, C. (1994). Lpaproscopic cholecystectomy in Leicester: an audit of 555 patients. *Ann. R. Coll. Surg. Rngl.*, **76**, 390–395.

[10] Soper, N. J. and Dunnegan, D. L. (1993). Laparoscopic cholecystectomy: experience of a single surgeon. *World J. Surg.*, **17**, 16–21.

[11] Wherry, D. C., Marohn, M. R., Malanoski, M. P., Hetz, S. P. and Rich, N. M. (1996). An external audit of laparoscopic cholecystectomy in the steady state performed in medical treatment facilicties of the Department of Defense. *Ann. Surg.*, **224**, 145–154.

[12] McGinn, F. P., Bell, N., Miles, S., Rew, D., Ozmen, M., Terci, G. and Uglow, M. (1995). Prospective randomised trial of laparoscopic and "mini" cholecystectomy. *Brit. J. Surg.*, **82**, 1374–1377.

[13] Majeed, A. W., Troy, G., Nicholl, J. P., Smythe, A. Reed, M. W. R., Stoddard, C. J., Peacock, J. and Johnson, A. G. (1996). Randomised, prospective, single-blind comparison of laparoscopic *versus* small-incision cholecystectomy. *Lancet*, **347**, 989–994.

[14] Barkun, J. S., Barkun, A. N., Sampalis, J. S., Fried, G., Taylor, B., Wexler, M. J., Goresky, C. A. and Meakins, J. L. (1992). Randomised controlled trial of laparoscopic *versus* mini cholecystectomy. *Lancet*, **340**, 1116–1119.

[15] McMahon, M. J., Russell, I. T., Baxter, S. N., Ross, S., Anderson, J. R., Morran, C. G., Sunderland, G., Galloway, D., Ramsey, G. and O'Dwyer, P. J. (1994). Laparoscopic *versus* minilaparotomy cholecystectomy: a randomised trial. *Lancet*, **343**, 135–138.

[16] Shea, J. A., Healey, M. J., Berlin, J. A., Clarke, J. R., Malet, P. F., Staroscik, R. N., Schwartz, J. S. and Williams, S. V. (1996). Mortality and complications associated with laparoscopic cholecystectomy. A meta-analysis. *Ann. Surg.*, **224**, 609–20.

[17] Cohen, M. M., Young, W., Theriault, M.-E. and Hernandez, R. (1996). Has laparoscopic cholecystectomy changed patterns of practice and outcome in Ontario? *Can. Med. Assoc. J.*, **154**, 491–500.

[18] Steiner, C. A., Bass, E. B., Talamini, M. A., Pitt, H. A. and Steinberg, E. P. (1994). Surgical rates and operative mortality for open and laparoscopic cholecystectomy in Maryland. *New Eng. J. Med.*, **330**, 403–8.

[19] Jatzko, G. R., Lisborg, P. H., Pertl, A. M. and Stettner, H. M. (1995) Multivariate comparison of complications after laparoscopic and open cholecystectomy. *Ann. Surg.*, **221**, 381–386.

[20] Dezeil, D. J., Millikan, K. W., Economou, S. G., Doolas, A., Ko, S.-T. and Airan, M. C. (1993). Complications of Laparoscopic Cholecystectomy: a national survey of 4,292 hospitals and an analysis of 77,604 cases. *Am. J. Surg.*, **165**, 9–14.

[21] Williams, L. F., Chapman, W. C., Bonau, R. A., McGee, E. C., Boyd, R. W. and Jacobs, J. K. (1993) Comparison of Laparoscopic with open cholecystectomy in a single center. *Am. J. Surg.*, **165**, 459–465.

[22] Clavien, P.-A., Sanabria, J. R. and Strasberg, S. M. (1992). Proposed classification of complications of surgery with examples of utility in cholecystectomy. *Surgery*, **111**, 518–526.

